# [Retracted] Metformin attenuates diabetic renal injury via the AMPK-autophagy axis

**DOI:** 10.3892/etm.2025.12957

**Published:** 2025-08-22

**Authors:** Tingli Sun, Jizhang Liu, Changying Xie, Jun Yang, Lijie Zhao, Jingbo Yang

Exp Ther Med 21:578, 2021; DOI: 10.3892/etm.2021.10010

Following the publication of this paper, it was drawn to the Editor’s attention by a concerned reader that certain of the western blot data shown in Figs. 2, 3 and 5, the Masson’s trichrome-stained murine renal sections in Fig. 1B, the immunohistochemical staining of the renal sections shown in Fig. 1D, the DHE-stained renal sections in Fig. 2A, and the TUNEL-stained renal sections in Fig. 2C were strikingly similar to data appearing in different form in other articles written by different authors at different research institutes that either had already been published elsewhere prior to the submission of this paper to *Experimental and Therapeutic Medicine*, or were under consideration for publication at around the same time.

In view of the fact that the abovementioned data had already apparently been published previously, the Editor of *Experimental and Therapeutic Medicine* has decided that this paper should be retracted from the Journal. The authors were asked for an explanation to account for these concerns, but the Editorial Office did not receive a reply. The Editor apologizes to the readership for any inconvenience caused.

